# Supplementary material for: Are carbon emissions trading and green financial instruments synergistic? -Comprehensive quantitative research based on content analysis
Source: PLoS One. 2024 Mar 7;19(3):e0298601. doi: 10.1371/journal.pone.0298601 (PMC10919601; doi:10.1371/journal.pone.0298601)
Supplement: S1 Data — (ZIP) [file pone.0298601.s001.zip › Data/地级市Z_F工作报告.zip!/╔╜╢1⁄2/╫═▓⌐╩╨/└┤╫╘╣1⁄2╓┌║┼í╛┬φ┐╦╩2╛▌═°í┐_2011.docx]

各位代表：

　　现在，我代表市人民政府，向大会作政府工作报告，请予审议，并请各位政协委员和其他列席会议的同志提出意见。

　　一、“十一五”时期经济社会发展回顾

　　“十一五”时期是我市发展史上极不平凡的五年。在中共淄博市委的坚强领导下，全市上下坚持以党的十七大和十七届三中、四中、五中全会精神为指导，深入贯彻落实科学发展观，积极应对国际金融危机等重大风险挑战，坚定不移走内涵发展的路子，突出加强结构调整，突出统筹城乡发展，突出改善民生维护稳定，充满激情，科学创业，胜利完成了“十一五”规划确定的各项目标任务，老工业城市转型取得重大进展，殷实和谐经济文化强市建设迈出坚实步伐。

　　“十一五”时期，我们始终坚持科学发展不动摇，全市综合实力迈上新台阶。过去五年，国内外环境的复杂性和重大风险挑战的严峻性历史罕见。面对极其复杂的国内外环境，尤其是面对国际金融危机的严重冲击，我们咬定发展不放松，奋力做好保增长、调结构、促改革、惠民生各项工作，快速扭转经济增速下滑趋势，保持了国民经济平稳较快发展的良好态势。2010年，全市完成规模以上固定资产投资1290.92亿元，社会消费品零售总额1005.68亿元，进出口总额67.23亿美元，分别是2005年的2倍、2.3倍和2.1倍，经济增长的协调性明显增强。与2005年相比，全市地区生产总值实现翻番，达到2866.75亿元，年均增长14.3%。境内财政总收入达到398.2亿元，其中地方财政收入162.4亿元，分别是2005年的2.48倍和2.53倍。金融机构存、贷款余额达到2470.56亿元、1686.13亿元，分别是2005年的2.24倍、2.29倍。城市居民人均可支配收入和农民人均纯收入达到21784元和9195元，分别是2005年的1.81倍和1.83倍。全市综合实力跨上了一个大台阶。

　　“十一五”时期，我们始终坚持下大气力转方式调结构，经济转型升级迈出新步伐。工业结构升级步伐加快。坚持一手抓传统产业改造升级，一手抓战略性新兴产业培育，提前完成“5年1500亿元”技改计划，地方骨干企业技术装备达到国际或国内先进水平的比重大幅度提高，一批传统产业实现脱胎换骨式的提升发展；启动实施新兴产业倍增工程，高新技术产业产值突破3000亿元，占规模以上工业总产值的比重达到38.2%，比2005年提高11个百分点以上，一批创新成长型企业快速发展壮大；中心城区东部化工区搬迁改造、南部建材区综合整治取得重要进展，产业布局调整优化初见成效。科技创新能力不断增强。省级以上企业研发机构达到172家、其中国家级8家，产业技术创新联盟建设实现新突破，东岳氯碱离子膜等一批重大高新技术项目实现产业化，中国名牌产品、中国驰名商标总数分别达到30个和43件，我市被确定为国家知识产权工作示范城市。服务业发展明显提速。一批外埠银行和著名连锁超市入驻淄博，文昌湖省级旅游度假区、中心城区城市综合体、淄博保税物流中心等一批重大项目加快推进，“齐风陶韵·生态淄博”旅游品牌影响力日益增强，服务业投资占比达到47%以上，服务业增加值比重比2005年提高5.7个百分点，达到34.7%。现代农业建设成效显著。粮食生产连续八年丰收，林果、畜牧、蔬菜等特色产业优势更加突出，无公害、绿色、有机农产品和地理标志产品达到221个，农产品质量安全水平显著提高，品牌农业、有机农业发展势头良好，都市农业顺利起步。

　　“十一五”时期，我们始终坚持统筹城乡发展，新型城镇化迈上新水平。按照“中心凸显、十字展开、组团发展”的思路，着力促进“四个层级”统筹协调发展，全市城镇化水平提高到62.5%。抓住承办省运会等重大机遇，建设了市体育中心、运动员公寓、淄博客运中心等一批标志性建筑，大力推进城区道路、管网改造和旧居住区综合整治，加强城市精细化管理，城市面貌焕然一新。组织实施济青高速南线沂源段、国道309张店立交、中心城区大外环、引太入张、南水北调淄博段、泰青威天然气管线淄博段等一大批基础设施建设工程，城市的承载力和辐射力显著增强。抓住扩内需、保增长的历史机遇，策划实施了总投资200亿元以上的“两区三村”改造建设工程，已累计开工项目291个，完成安置房面积874万平方米，7万多户城乡居民陆续乔迁新居，促统筹、惠民生、拓空间、带产业等多重功效日益显现。深入实施农村殷实小康十大工程，社会主义新农村建设取得显著成就。全面完成大中小型水库除险加固，五年新建改造农村公路2320公里，3008个行政村的群众喝上了自来水，水、电、路、通讯、广电“村村通”和城乡一体的公共服务水平不断提高。

　　“十一五”时期，我们始终坚持加强节能减排和环境保护，生态文明建设取得新成效。坚持把节能降耗作为关键，把环境保护作为“命门”，以前所未有的力度狠抓工作推进和落实。严格目标责任约束，依法淘汰落后产能，切实加强节能减排技术改造，大力发展循环经济。先后淘汰水泥落后产能1100万吨、钢铁落后产能302万吨、建陶落后产能7750万平方米，关停“土小”企业3800多家。组织实施两轮“碧水蓝天行动计划”，完成环保投入260亿元，是“十五”期间的1.6倍。深度治理大气污染，空气质量良好率显著提高。新改扩建14座城镇污水处理厂，出水水质全部达到一级A排放标准，全市8条主要河流水质全面达到恢复鱼类生长的要求。建成市垃圾焚烧发电厂，城市生活垃圾全部实现无害化处理。全面提高大环境绿化水平，五年新增林地45万亩、园林绿地2000公顷，全市森林覆盖率达到35%，建成区绿化覆盖率达到42.2%，成功创建为国家园林城市和全国绿化模范城市。组织实施四宝山以及孝妇河、猪龙河、沂河、淄河等重点区域、重点流域生态修复工程取得重大成果。深入开展城乡环境综合整治，秸秆全面禁烧及综合转化利用工作成果不断巩固扩大，循环农业示范市建设取得明显成效。现在可以肯定地说，我们能够全面完成“十一五”节能减排各项目标任务，让淄博天更蓝、地更绿、水更清、气更爽的目标正在加快变为现实！

　　“十一五”时期，我们始终坚持用改革的办法破解发展难题，改革开放实现新突破。扎实推进重要领域和关键环节改革，着力消除制约老工业城市转型发展的体制机制障碍。市县政府机构改革基本完成，乡镇布局调整和机构改革稳步实施，扩权强镇改革顺利启动，区县域发展战略日益完善，经济社会发展活力明显增强。企业改革不断深化，创造性地组织实施一系列重大资产重组，上市公司历史遗留问题全部解决，股权分置改革全面完成，“淄博板块”健康发展壮大，五年新增上市公司8家，直接融资额达126亿元。抓住国家建立多层次资本市场的历史机遇，设立齐鲁股权托管交易中心，全省性股权交易市场建设迈出关键步伐。基本完成集体林权制度改革主体任务，其他各项改革取得积极成效。有效应对国际金融危机冲击，开放型经济水平不断提高。“十一五”期间，实际利用外资23.8亿美元，完成出口162亿美元，分别是“十五”时期的1.62倍和2.7倍，中海外、华润、茂业、PPG等一大批国内外著名企业进入淄博，宏达矿业成功收购秘鲁邦沟铁矿。成功举办了五届陶博会和新材料技术论坛，“新材料名都”和“淄博陶瓷·当代国窑”品牌影响力不断扩大。区域经济交流合作取得新成果，援川、援藏、援疆等工作成效显著。

　　“十一五”时期，我们始终坚持保障和改善民生，社会与民生建设取得新成果。各级财政用于民生建设的投入大幅增加，各项民生政策得到全面落实。实施积极的就业政策，五年累计新增城镇就业59万人次，农村劳动力转移就业39万人次。覆盖城乡的社会保障体系初步形成，保障水平逐步提高，城镇五项社会保险在全国同类城市中率先实现市级以上统筹，国有(集体)破产关闭企业退休人员医疗保险问题得到全部解决，新型农村合作医疗、新型农村养老保险实现全覆盖，城乡低保不断提标扩面，多层次住房保障体系逐步建立。五年来，我们全面实行了真正免费的义务教育，初步建立了从小学到大学的家庭经济困难学生资助体系；启动了医药卫生体制改革，政府办基层医疗机构实现国家基本药物零差率销售；组织实施了中小学布局规划调整、危房改造、标准化建设以及县乡卫生院、村卫生室改造提升等一大批民生和社会建设重点工程，突发公共卫生事件应急机制不断健全完善，低生育水平保持稳定，城乡教育、医疗条件显著改善。成功协办和承办第11届全运会、第22届省运会、省第8届残运会、亚青赛等重大体育赛事，全民健身深入开展，竞技体育连创佳绩。群众性精神文明创建活动蓬勃深入，文化事业和文化产业繁荣发展，被命名为创建全国文明城市工作先进市和全国未成年人思想道德建设先进市。国防动员和民兵预备役工作扎实有效，双拥工作实现“六连冠”。固本强基维稳工程深入实施，“平安淄博”建设成效显著，安全生产形势保持稳定，食品药品安全和质量监管不断加强，应急管理体制日益完善，荣获全国社会治安综合治理优秀市称号。妇女儿童、慈善、残疾人、红十字、仲裁、老龄、民族宗教、外事、侨务、对台、史志、档案、人防、防震减灾、气象、统计、新闻出版、广播电视等各项事业都取得了新成绩、实现了新发展。

　　在推动经济社会又好又快发展的同时，我们始终高度重视转变政府职能、加强自身建设，法治政府、责任政府、阳光政府和服务型政府建设取得新成效。深入推进依法行政，不断完善科学化、民主化、法治化决策机制，深化行政审批制度改革，全面推行政务公开，严格规范行政执法行为，加大行政问责力度。自觉接受人大及其常委会的监督和政协的民主监督，虚心听取各民主党派、工商联和社会各界的意见，认真办理人大代表建议和政协委员提案。严格落实党风廉政建设责任制，强化行政监察和审计监督，严肃查处违法违纪案件。切实加强公务员队伍建设，大力弘扬认真专业务实政风，不断健全推进落实的工作机制，行政效能和服务水平进一步提高。刚刚过去的2010年，全市上下认真贯彻落实党的十七大和十七届五中全会精神，统筹推进保增长、转方式、调结构、惠民生各项工作，胜利完成了市十三届人大三次会议确定的各项目标任务。全市地区生产总值比上年增长13.7%，地方财政收入增长26.1%，规模以上固定资产投资增长22.6%，社会消费品零售总额增长18.8%，外贸进出口与实际利用外资分别增长39.1%、11.8%，城市居民人均可支配收入和农民人均纯收入分别增长13%、14.75%，为“十一五”发展画上了一个圆满的句号。

　　各位代表！过去五年的发展历程波澜壮阔。在中共淄博市委的坚强领导下，我们积极作为、化危为机，坚决贯彻落实中央和省委、省政府一系列重大决策部署，积极应对国际金融危机冲击，谱写了在逆境中奋进、在转型中发展的壮丽篇章；我们万众一心、顾全大局，以顽强的作风和一流的工作，出色完成了支援北川灾后重建以及“4·28”救援处置等重大急难险重任务，彰显了淄博城市精神，为淄博和全省人民增了光添了彩；我们全力以赴、众志成城，成功举办了第22届省运会、亚青赛等一系列重大赛事，向省委、省政府和全省人民兑现了举办一届有特色、高水平、精彩圆满体育盛会的庄严承诺，充分展示了淄博科学发展、和谐发展的成就，极大地提振了全市人民的自信心和自豪感。这些大事、盛事、难事，必将在淄博经济社会发展史上留下浓墨重彩的一页！

　　过去的五年，是老工业城市加速转型发展的五年，是淄博新的竞争优势明显增强的五年，是人民群众得到更多实惠的五年，是我们在建设殷实和谐经济文化强市的道路上阔步前进的五年。如今的淄博，经济繁荣，社会和谐，政通人和，人心思上。这样的大好局面，得益于党中央、国务院和省委、省政府的科学决策和亲切关怀，胡锦涛总书记、温家宝总理亲临我市视察，给全市人民极大鼓舞和有力鞭策。这样的大好局面，得益于市委的正确领导，得益于市人大、市政协和社会各界的大力支持，得益于全市人民团结拼搏、励精图治、奋发努力。在此，我代表市人民政府，向全市人民，向各民主党派、工商联、各人民团体和社会各界人士，向驻淄部队、武警官兵、政法干警和中央、省驻淄单位，向海内外所有关心支持淄博发展的同志们、朋友们，表示衷心的感谢和崇高的敬意！

　　在充分肯定成绩的同时，我们也清醒地看到，我市经济社会发展还面临一些困难和问题，主要是：经济结构性矛盾依然比较突出，自主创新能力和产业竞争力还不够强，转变发展方式任务繁重；资源环境和发展空间约束增强，节能减排压力依然较大，推动可持续发展面临新的挑战；统筹城乡一体化和促进区域协调发展任务艰巨，保障和改善民生财政压力很大。同时，面对科学发展的新形势新要求，政府职能还需进一步转变，依法行政的能力还需进一步提高，作风效能建设还需进一步加强。对这些困难和问题，我们高度重视，要采取更加扎实有效的措施，努力加以解决。

　　 二、“十二五”时期经济社会发展的总体思路和目标任务

　　从今年开始，已进入第十二个五年规划时期。根据党的十七大、十七届五中全会精神和市委十届十一次全会精神，市政府编制了《淄博市国民经济和社会发展第十二个五年规划纲要(草案)》。《纲要(草案)》及《说明》已印发大会，请一并予以审议。

　　“十二五”时期是全面建设小康社会的关键时期，是深化改革开放、加快转变经济发展方式的攻坚时期。今后五年，我们要高举中国特色社会主义伟大旗帜，以邓小平理论和“三个代表”重要思想为指导，深入贯彻落实党的十七大和十七届三中、四中、五中全会精神，以科学发展为主题，以加快转变经济发展方式为主线，以富民强市为目标，进一步坚持内涵发展，突出加强结构调整，突出统筹城乡发展，突出改善民生维护稳定，深化改革开放，着力推动淄博老工业城市新的转型发展，不断开创殷实和谐经济文化强市建设新局面。

　　——以科学发展为主题，就是始终坚持发展是硬道理的本质要求，聚精会神搞建设，一心一意谋发展，更加注重以人为本，更加注重全面协调可持续发展，不断增创新优势，实现新跨越。

　　——以加快转变经济发展方式为主线，就是把加快转变经济发展方式贯穿到“十二五”发展全过程和经济社会各领域，痛下决心、坚定不移打好转方式调结构这场硬仗，切实做到在发展中促转变、在转变中谋发展。

　　——以富民强市为目标，就是坚持从人民群众的根本利益出发谋发展、促发展，坚持经济与文化融合发展，强市与富民有机统一，切实做到发展为了人民，发展依靠人民，发展成果由人民共享。

　　——进一步坚持内涵发展，突出加强结构调整，突出统筹城乡发展，突出改善民生维护稳定，就是将这一符合科学发展观要求、符合淄博发展阶段性特征的总体工作思路长期坚持下去，努力走出一条具有淄博特色的科学发展新路子。

　　——着力推动淄博老工业城市新的转型发展，就是坚持转方式、调结构、促增长有机统一，坚持规模、质量、效益有机统一，坚持产业布局、城镇建设、生态保护和谐统一，全面提高内涵发展、科学发展水平。

　　根据这一指导思想，综合考虑未来发展趋势和条件，《纲要(草案)》提出了“十二五”时期的主要发展目标，主要包括五个方面：一是经济保持平稳较快发展，地区生产总值年均增长12%左右，到2015年力争突破5000亿元；地方财政收入年均增长15%左右，到2015年突破300亿元。二是结构调整取得重大进展，服务业增加值占地区生产总值的比重提高到43%，高新技术产业产值占规模以上工业总产值的比重每年提高1个百分点以上，全面完成节能减排任务，初步建立起以战略性新兴产业和现代服务业为主导的产业体系。三是统筹城乡发展迈出更大步伐，“四个层级”的新型城镇化格局基本形成，城镇化率提高到68%，生态和谐宜居的城市特色更加彰显。四是群众生活更加殷实富裕，城市居民人均可支配收入和农民人均纯收入年均分别增长12%和13%左右，实现城乡群众收入与经济发展同步增长。五是社会事业协调发展，社会管理明显加强，社会更加和谐稳定。

　　各位代表！深入分析国内外发展形势，“十二五”时期仍然是可以大有作为的重要战略机遇期，我们既面临难得的历史机遇，也面临诸多可以预见和不可预见的风险与挑战。我们要切实增强机遇意识和忧患意识，科学把握发展规律，加快转变发展方式，着力推动淄博老工业城市新的转型发展，实现科学发展、富民强市新跨越。这是我市社会主义现代化建设承前启后、继往开来的重大历史使命，是全市人民过上更加美好生活的新期待新要求。我们坚信，有市委的坚强领导，有“十一五”发展打下的良好基础，有广大人民群众的信任和支持，只要我们同心同德、奋发进取，充满激情、科学创业，就一定能够战胜各种艰难险阻，把“十二五”美好蓝图变为现实，谱写殷实和谐经济文化强市建设的新篇章！

　　 三、2011年经济社会发展的目标任务和工作重点

　　2011年是建党90周年，也是“十二五”开局之年，做好今年工作具有十分重要的意义。考虑到与“十二五”发展目标相衔接，今年经济社会发展的主要预期目标建议为：地区生产总值增长12%左右，地方财政收入增长15%左右，规模以上固定资产投资增长20%左右，社会消费品零售总额增长18%左右，外贸进出口与实际利用外资均增长12%左右，城市居民人均可支配收入和农民人均纯收入分别增长12%和13%左右，人口自然增长率控制在2.6‰以内，城镇登记失业率控制在4%以内，居民消费价格涨幅控制在4%左右。万元生产总值能耗、二氧化硫、化学需氧量、氮氧化物、氨氮排放量削减目标，按照省下达的“十二五”总量控制任务分解到各年度，作为约束性指标确保完成。

　　重点抓好以下五个方面的工作：

　　(一)努力保持经济平稳较快发展。深入把握积极稳健、审慎灵活的宏观经济政策取向，贯彻落实好积极的财政政策和稳健的货币政策，始终把保持经济平稳较快发展，作为一项长期性、全局性的重要战略任务，牢牢抓在手上。

　　抓好即期经济运行。加强对重要生产要素和生活资料的组织协调，确保城乡居民生活和企业正常生产的需求。积极帮助企业加大市场开拓力度，特别要研究利用好国家拉动内需的政策，扩大我市大宗产品的国内市场份额。加强市场与价格调控监管，保障重要生活必需品供应。及时采取临时价格补贴等措施，对相关困难群体进行补助，加快建立社会救助和保障标准与物价波动挂钩的联动机制。

　　促进投资、消费、出口协调拉动。一是保持有效投资的适度较快增长。充分发挥投资拉动经济增长的主导作用，把握投资重点，优化投资结构，促进投资稳定增长。加快推进扩内需、保增长结转项目建设，确保按期竣工达效。健全重大项目推进机制，完善建设条件，强化要素支撑，启动实施一批“十二五”规划重点项目。二是更加注重发挥消费对经济增长的拉动作用。认真落实中央和省关于收入分配制度改革的要求，努力实现居民收入增长与经济发展同步、劳动报酬增长与劳动生产率提高同步，增强居民消费能力。完善城乡流通网络，加快构建现代流通体系，不断优化消费环境，促进消费结构优化升级。三是稳定和拓展外需。加快出口结构转型升级，大力培育机电、医药、纺织、轻工等特色产业出口基地，扩大机电产品和高新技术产品出口。支持高新区等服务外包产业聚集区发展，扩大服务贸易规模。增加能源资源性产品、先进技术装备以及关键零部件进口。继续鼓励具有比较优势的企业“走出去”投资办厂，建设境外资源生产供应基地，扩大对外工程承包，拓展经济发展空间。

　　做好财税金融工作。强化效益优先理念，加强财政、信贷、产业、土地和节能、环保政策的协调配合，加大对优质财源项目的支持力度，合力推动转方式、调结构、增财源。依法加强税收征管，加大综合治税和非税收入管理力度，提高国有资产和政府资源运营收益。优化支出结构，严格控制一般性支出，集中财力保重点、保民生。进一步优化金融生态环境，完善政银企协作机制，支持金融机构创新金融产品、优化信贷投向，更好地为保发展、转方式服务。加快地方金融体系建设，支持地方金融机构创新发展、做大做强。

　　(二)推动结构调整实现新突破。经济结构的战略性调整是推进发展方式转变的主攻方向，是一项长期而艰巨的重要任务，必须以更大的决心和力度加以推进，务求取得实质性进展。

　　着力调整优化工业结构。坚持改造提升传统产业与培植壮大新兴产业“双轮驱动”，切实提高淄博工业整体素质和竞争力。一是加快传统产业改造升级。启动实施新一轮技改计划，建立重大装备改造目录，逐年组织实施。每年重点扶持30个左右设备投资额在2亿元以上，规模、效益指标在国内同行业领先的重点技术改造项目，加快提升传统产业的技术装备和工艺水平。年内力争完成企业技改投资670亿元以上。通过技术改造，着力培植龙头企业，培育名牌产品，拉长产业链，提高产业集中度，壮大优势产业集群。深入推进工业化、信息化“两化融合”，在重点行业培植15家信息化示范企业，扶持一批“两化融合”项目，以信息化促进传统产业改造升级。抓住时机引进更多的行业领军企业、国内外前沿技术和优质资本，做足做活“引进优质增量，盘活提升存量，扩大优化总量”这篇大文章。二是大力培育战略性新兴产业。积极实施新兴产业倍增工程，三年内筹措新兴产业专项资金3亿元，全力支持新材料、新能源、新医药等一批重大核心关键技术研发及产业化项目，加速形成技术与规模优势，提高核心竞争力，争取到2012年，新兴产业产值在2009年基础上翻一番。壮大创新成长型企业群体，增强对新兴产业的拉动作用。建立支持新兴产业跨越发展的体制机制，对重大革命性技术成果以及能够带来巨大市场效益的产业化项目，从土地、融资、人才、服务等各个方面研究采取优惠政策，全力以赴予以保障支持。

　　加快服务业提质增效。坚持壮大规模与提高质量并重，力争全市服务业增加值占比提高2个百分点左右，服务业对就业和地方财政的贡献明显提高。一是着力构建服务业跨越发展的优质载体。立足优势、突出特色，编制实施全市重点服务业发展规划，加快鲁中物流“旱码头”、全省性股权交易市场和国内外知名旅游品牌城市建设步伐。加大政策倾斜和要素保障力度，着力扶持一批既与现代制造业密切相关、又能有效提升城市功能、显著增加地方财源的高端生产性服务业和生活性服务业项目。集中力量推进文昌湖旅游度假区建设，争取用五年左右时间，将这一区域建设成为统筹城乡示范区、高端服务业聚集区，带动全市服务业规模扩张和业态提升。二是完善政策体系。认真落实省政府关于支持服务业跨越发展的30条政策，结合我市实际，研究制定配套措施，为服务业发展创造良好环境。进一步推动服务业对外开放，积极引进优质资本和著名品牌，带动服务业转型升级。三是加快推进大型生产企业剥离非核心业务。落实优惠政策，加快推进重点制造业企业非核心业务的剥离工作，培育一批面向生产、独立运营、单独纳税的生产性服务业群体。

　　加快发展精准农业和都市农业。积极推进农业转方式调结构，加快构建高产、优质、高效、生态、安全的现代农业产业体系，促进农业持续增效、农民持续增收。一是提高粮食综合生产能力。深入开展粮食高产创建活动，加强桓台、高青、临淄“全国新增千亿斤粮食生产能力规划县”以及沂源、临淄全国旱作农业示范基地项目建设，改造提升百万亩优质专用粮食基地。二是启动实施蔬菜、畜牧、果业、苗木花卉四大产业振兴计划。整建制推进沂源、博山和淄川东南部有机农产品生产区建设，以10万头山东黑牛产业化工程为龙头，加快建设高青黄河三角洲高效生态畜牧区，推进农业产业化、标准化、品牌化。三是大力发展都市农业。注意搞好与城建规划、土地利用规划、生态建设规划的有机衔接，突出抓好中心城区核心都市农业项目区和百家市级示范园区建设，丰富都市农业内涵，提高农业综合效益。四是进一步巩固农业基础地位。全面落实各项强农惠农政策，拓宽农民增收渠道。抓住用好国家启动新一轮水利设施建设、省里启动现代水利示范省建设的机遇，积极争取中央和省投资，加紧规划建设一批水利项目。今年再解决8.5万农村人口饮水安全问题。继续深化农村集体林权制度改革，积极稳妥推进农村土地承包经营权流转市场建设，大力发展农民专业合作社，增强农业和农村发展活力。

　　去年入冬以来，我市遭遇历史罕见的特大干旱，目前旱情还在进一步发展。我们要切实加强组织领导，动员全社会力量加大资金、科技、物资投入，确保大旱之年农业丰收、人畜饮水安全，坚决打好抗旱夺丰收、保发展、保民生这场硬仗。

　　(三)强化转方式调结构的有效支撑。打好转方式调结构这场攻坚战，需要切实强化关键因素、重要条件的有力支撑。为此，必须在以下五个方面狠下功夫：

　　坚定不移抓好节能减排。进一步健全节能减排长效机制，强化节能减排目标责任考核，根据省下达我市的“十二五”和2011年节能减排目标，逐级分解落实到各区县、各有关部门和重点企业，加强督查，确保不折不扣地完成。加大淘汰落后产能力度，坚决关停能耗高、污染重、不具备安全生产条件的“土小”企业，确保完成省下达给我市的淘汰落后产能计划。加大对节能减排新技术、新工艺、新产品以及发展循环经济重点企业的扶持力度，加快重点装备节能减排技术改造，积极发展节能环保产业，加强公交、建筑以及公共机构等重点领域节能工作，积极稳妥地推进资源性产品价格和环保收费改革，加快构建低碳产业体系和绿色生活方式。把优化能源消费结构摆上重要位置，建设中石油沧淄线与泰青威天然气管线连接线工程，稳步推进清洁能源替代工作。

　　切实改善城乡生态环境。以创建国家环保模范城市为目标，全面提升城乡环境质量。一是加大污染治理力度。深度实施大气污染治理再提高工程，积极推进小电厂整合、大电厂替代发电工作，有效防治二氧化硫、粉尘、烟尘和扬尘污染。新建扩建改造一批城镇污水处理厂，加大城区雨污管网改造力度，污水处理能力提高到85万吨/日。继续抓好重点流域综合整治，确保全市主要河流水质稳定达标。大力加强水源地保护、农村面源污染治理，尽快完善城乡垃圾一体化处理体系，切实保障环境安全。大力推广农村沼气，巩固秸秆禁烧和转化利用成果。加大结构减排、管理减排力度，严厉打击环境违法行为。二是扎实推进生态建设。重点实施“两库三河一山一湿地”生态建设工程、“清水润城”工程和“森林围城”工程，高标准完成中心城区大外环绿化等重点项目，全年完成造林10万亩，新增园林绿地280公顷以上，加快创建国家生态园林城市。

　　着力增强创新发展能力。抓住我省开展国家技术创新工程试点的机遇，进一步完善创新体系。一是大力推进创新平台建设。组织开展“技术中心提升计划”，支持东岳膜工程研发中心建设，提升氟硅材料和医疗设备两大技术创新联盟的运作水平，加快组建化工行业技术创新联盟，提高集成创新能力。支持高新区创建国家创新型科技园区，切实发挥其辐射、带动功能。二是围绕提升产业核心竞争力，深化产学研合作。加强技术攻关，组织实施一批重大技术创新项目，重点抓好与山东理工大学等高等院校、科研院所在新能源汽车、超级电容、集成电路、大功率磁悬浮电动机等方面的联合攻关，力求取得实质性突破。三是优化创新环境。以建设国家知识产权工作示范市为契机，提高知识产权创造、运用和保护工作水平。设立市政府企业管理创新奖，推动企业管理创新。坚持人才优先、以用为本，加强高层次人才和高技能人才队伍建设，激发全社会创新活力。

　　大力推进集约发展。当前，我市发展面临的空间约束日益强化，这是影响可持续发展的重大瓶颈制约，必须高度重视优化产业布局、促进集约发展。一是提高园区集约集聚发展水平。切实加强新一轮城市总体规划、土地利用总体规划和年度计划、产业布局规划之间的有效衔接，以现有省级以上园区为主要载体，完善园区考核办法，创新园区利益分配机制，激励企业向园区集中，努力把重点园区建设成为高端高质高效项目和产业的聚集区。二是提高土地节约集约利用水平。深化国土资源管理改革试点，强化用地规划管控，加大存量土地盘活力度，优先保障重点项目建设用地。积极稳妥推进农村土地综合整治，进一步将土地综合整治与增减挂钩和农村住房建设有机结合起来，在尊重农民意愿、维护农民合法权益的前提下，加大启动资金投入，加快工作推进，提高规范整治水平。

　　创新投融资体制。政府投资要更加注重讲求收益和效率，发挥“四两拨千斤”的引导带动功能。切实放宽市场准入，进一步激活民间投资。毫不放松地抓好招商引资工作，突出抓好产业招商，加大股权并购、境外上市力度，扩大招商引资规模，优化利用外资结构，显著提高新兴产业和现代服务业利用外资比重。大力培育上市资源，推动具备条件的企业尽快实现上市，为转方式调结构提供坚实支撑。按照立足鲁中、辐射全省的要求，支持齐鲁股权托管交易中心健康快速扩容，全面提升监管运营水平，更好地发挥全省性股权交易市场的功能。

　　(四)扎实推进城乡统筹发展。坚持把统筹城乡发展作为转变经济发展方式的重要依托，科学把握新型城镇化发展趋势，加快生态和谐宜居城市建设，提高统筹发展水平。

　　进一步强化规划引控。贯彻落实国家主体功能区战略，深度对接全省“一蓝一黄”重大区域发展规划，抓紧编制完善全市主体功能区规划，明晰优化开发区、重点开发区、限制开发区和禁止开发区，形成全面、协调、可持续的空间开发建设格局。启动实施全市新一轮土地利用总体规划和城市总体规划，突出抓好“四个层级”控制性详细规划的编制，按照“六个一体化”要求，编制完善产业、生态、基础设施、公共服务等专项规划，实现发展目标、国土指标与规划坐标的有机统一。全面提升城市设计水平，进一步彰显城市特色，提升城市形象。

　　着力提升城镇功能。坚持“中心凸显、十字展开、组团发展”，协调推进新城区建设和老城区改造，全面提高城市现代化水平。一是加快新城区建设步伐。集中力量推进市文化中心建设，加快提升新城区商务、行政、文体功能，高质量建设新城区水系，启动中心城区百栋高层建筑建设工程，进一步推升中心城区建设发展水平。二是加大旧城改造力度。探索对老城区实行一个开发主体、连片综合开发新模式，集中推进中心城区城市综合体建设，打造功能现代、辐射力强的高端服务业聚集区。加大老旧居住区综合整治力度，基本完成主要道路两侧建筑物立面整治和背街小巷环境整治，进一步改善城市面貌。三是全面完成“两区三村”改造建设任务。如期完成三年改造建设计划，确保群众和谐还迁，兑现党委、政府向广大群众的郑重承诺。同时，与中心镇改革发展试点有机结合，加强中心镇、中心村规划建设，培育一批有一定产业基础、辐射带动能力强、特色鲜明的经济强镇、文化名镇和区域重镇。四是加强重要基础设施建设。组织实施一批公路交通、城区路网改造和农村公路建设重点工程，实现中心城区大外环东、北、西三面贯通。深化公交优先战略，创造安全快捷、低碳环保的出行环境。积极推进轻轨交通规划设计，推动晋豫鲁铁路通道沂源段、寿平铁路桓台段顺利建设。加快南水北调淄博段建设步伐。完善城乡一体化供水体系，确保城乡居民饮水安全。

　　提高城市管理水平。强化规划执法，确保规划的严肃性和权威性，坚决查处各类违规违章建设。加强市级数字化城市管理平台建设，构建网格化、即时性、全覆盖的城市管理长效机制，提高城市管理的精细化、规范化、科学化水平。深入实施城乡环境综合整治，抓好村镇硬化、绿化、净化、亮化，进一步改善城乡环境质量。

　　(五)加强社会建设切实保障民生。大力加强社会建设与管理创新，建立健全公共服务体系。全面落实国家和省有关保障和改善民生的决策部署，调整优化财政支出结构，将更多的财力投向民生支出，在改善民生上多办实事。

　　促进就业稳定增长。实施就业优先发展战略，多渠道开发就业岗位，最大限度增加就业容量。落实各项就业扶持政策，推进“三支一扶”、“服务社区计划”等基层就业项目，突出抓好高校毕业生等重点群体就业工作。加快规划建设统一规范的市级人力资源市场，完善职业介绍和培训补贴政策，加强创业孵化基地建设，提高劳动者就业、创业能力。力争年内新增城镇就业7.6万人，新增农村劳动力转移就业6.2万人。

　　健全社会保障体系。一是提高社会保障水平。根据省政府要求落实好市县配套资金，按期实现国家新农保全覆盖。选择有条件的区县开展城镇居民养老保险试点。加快建立社会保险待遇与经济发展、居民收入增长相联系的正常调整机制，稳步提高社会保障水平。较大幅度提高城乡低保、城镇居民医保、新农合财政筹资和公共卫生服务人均标准。继续提高企业退休人员养老金水平，提高失业保险金标准，调整工伤人员伤残津贴、护理费和供养亲属抚恤金标准。落实集中和分散抚养孤儿基本生活费财政支付政策。二是推进保障性住房建设。大规模推进公租房等保障性安居工程建设，多渠道增加保障性住房投入，积极开展公积金支持保障性住房建设试点。通过新建、改建等方式新增各类保障性住房8700套，新增廉租住房补贴500户。

加快发展社会事业。认真组织实施全市中长期教育改革和发展规划纲要，全面推进素质教育，加快普及学前三年教育，大力发展职业教育，积极开展教育改革试点，努力在促进教育公平的基础上增加优质教育资源。提高农村中小学生均公用经费补助标准和寄宿生生活费补助标准，提高各类高校和职业学校的生均拨款水平，提高高校助学金标准。继续深化医药卫生体制改革，着力抓好基本药物制度建设和公立医院改革试点，尽快启动市医疗中心规划设计工作，加快构建新型城市卫生服务体系和农村三级医疗卫生服务网络。健全激励和制约并重的利益导向机制，稳定低生育水平。健全食品药品监管网络，提高食品药品质量安全水平。抓好全市文化体制改革和文化产业振兴大会决策部署的落实，繁荣发展文化事业，加强公共文化服务体系建设，加快把文化产业打造成为国民经济新的支柱性产业。促进妇女儿童、老龄、残疾人、慈善、红十字等各项事业健康发展。努力争创全国文明城市。加强国防动员、民兵预备役和人民防空建设，深入推进双拥共建，努力实现“七连冠”。统筹群众体育、竞技体育、体育产业协调发展，做好第七届全国城运会协办工作。

　　推进社会管理创新。适应社会结构加快变动、利益格局深刻调整的新形势，科学整合社会管理资源，创新社会管理方式。深入实施第三个社区建设三年规划，增强社区的管理和服务功能。加强流动人口、网络虚拟社会和社会组织管理服务工作。大力实施固本强基维稳工程，推动“法治淄博”建设，以群众工作统揽信访工作，着力解决涉及群众利益的突出问题。完善社会治安防控体系，依法防范和严厉打击各类违法犯罪活动，不断提高人民群众的安全感和对社会治安的满意度。扎实开展“安全生产基层基础深化年”活动，坚决遏制较大以上安全生产事故。加强突发事件应急管理，提高预防预警和应急处置能力。

　　四、加强政府自身建设

　　面对“十二五”时期经济社会环境的新变化和人民群众的新期待，必须着力转变政府职能，切实提高依法行政水平和行政效能，加快建设法治政府、服务政府。

　　(一)深化行政管理体制改革。深入推进扩权强县、扩权强镇改革，巩固发展市县政府机构改革和乡镇机构改革成果，搞好经济发达镇行政管理体制改革和中心镇发展改革试点，增强区域发展内生动力。积极稳妥地推进事业单位分类改革。进一步减少和规范行政审批，强化各级行政服务中心“一站式”服务功能，着力推进行政审批“两集中、两到位”和建设项目联合审批改革，加强电子政务建设，推动政府管理与服务创新。

　　(二)提高依法行政水平。更加注重加强制度建设，强化对行政权力运行的监督和制约，加快建设法治政府。健全重大行政决策规则，完善风险评估机制，推进决策的科学化、民主化、法治化。进一步规范行政执法，严格依照法定权限和程序行使权力、履行职责，严格规范行政裁量权行使，避免执法的随意性。加强行政复议工作，依法化解社会矛盾纠纷。自觉接受人大及其常委会的监督、政协的民主监督，高度重视舆论监督，依法保障人民群众监督政府的权力。强化行政监察和审计监督，严格行政问责，对有令不行、有禁不止、行政不作为、乱作为、失职渎职等行为，依法依纪严肃追究有关责任人直至主要行政负责人的责任，坚决做到有错必纠、有责必问、从严治政。完善依法行政考核制度。全体政府工作人员特别是领导干部要带头学法、尊法、守法、用法，做依法行政的表率。

　　(三)大力转变工作作风。面对复杂多变的经济形势和艰巨繁重的工作任务，各级政府及其部门要密切关注、准确把握宏观经济政策变化，更加善于发现机遇、抓住机遇、用好机遇，善于化挑战为机遇、变压力为动力，牢牢掌握工作主动权。进一步深化作风效能建设，把认真专业务实的要求融入到推动改革发展的具体工作中，体现到一个个工作项目上，狠抓落实，务求实效。健全完善与转变经济发展方式、促进内涵发展相适应的工作考核和绩效评价体系，树立正确导向，严格考核奖惩，确保各项任务目标顺利实现。

　　(四)加强廉洁勤政建设。深入推进反腐倡廉制度创新，严格执行党风廉政建设责任制，加大查办违纪违法案件工作力度，坚决查处和纠正损害群众利益的不正之风，维护社会公平正义，提高政府公信力。弘扬艰苦奋斗、勤俭节约的优良作风，加强节约型机关建设，各级行政性支出做到零增长，努力降低行政成本，把有限的资金和资源更多地用在发展经济和改善民生上。全体政府工作人员特别是领导干部，要牢记为人民服务的根本宗旨，严格执行廉洁自律各项规定，加强修养，自警自励，秉公用权，以廉洁勤政的实际行动取信于民，树立和维护政府的良好形象。

　　各位代表，我们已经踏上“十二五”改革发展的新征程。站在新的历史起点上，我们倍感肩负的使命重大而光荣。让我们更加紧密地团结在以胡锦涛同志为总书记的党中央周围，以邓小平理论和“三个代表”重要思想为指导，深入贯彻落实科学发展观，在中共淄博市委的坚强领导下，埋头实干，锐意进取，为夺取全面建设小康社会新胜利而努力奋斗！
